# Characterizing force capability and stiffness of hip exosuits under different anchor points

**DOI:** 10.1371/journal.pone.0271764

**Published:** 2022-08-04

**Authors:** Jihun Kim, Junyoung Moon, Sungjin Park, Giuk Lee

**Affiliations:** Departement of Mechanical Engineering, College of Engineering, Chung-Ang University, Seoul, Republic of Korea; Istituto Italiano di Tecnologia, ITALY

## Abstract

Exosuits have been broadly researched owing to their benefits from soft and deformable nature. However, compared to exoskeletons, the exosuits have disadvantages in that the deformation of suit and human tissue can cause dissipation, leading to low force transfer efficiency. In this study, we explore the force capability and human-suit stiffness depending on the anchor point positions of the exosuit, introducing a better understanding of exosuit design. We found the relationships between the anchor point position and the force capability, and the anchor point position and the human-suit stiffness by conducting human subject experiments. When the distance between the anchor point of the waist belt and the anchor point of the thigh brace increased, the force capability increased, whereas the human-suit stiffness decreased. Also, statistical analyses are implemented to verify significant differences according to the anchor point position with a 5% significance level. Moreover, we discuss why the capability of force transmission and the human-suit stiffness differ depending on the anchor point positions. The force capability differed with anchor point positions because of the change in the effective cable stroke. Additionally, the force capability changes nonlinearly owing to the body curve as the condition level of the anchor points changes. The human-suit stiffness is affected by the interference of the body when the assistive force is transmitted through the cable. Characteristics of the force capability and human-suit stiffness model can be used to optimize the performance of existing exosuit or to serve a valuable guide of design a new exosuit when the exosuit needs to maximize the force capability or stiffness.

## Introduction

Exosuits, a form of wearable robots, have drawn significant attention from researchers owing to their high utility [1–8]. An exosuit is a garment that comprises soft textiles, and thus, it does not hinder natural body motion and is simpler than an exoskeleton comprising a rigid structure with solid materials [9–11]. Owing to its lightweight and low-profile design, an exosuit has fewer adverse effects due to inertia during movements. Thus, it exhibits the advantage that the metabolic penalty is smaller than that of the exoskeleton with heavier mass [12–15].

Although exosuits exhibit several advantages, which are necessary for wearable robots, they also have some limitations. For instance, it is difficult to obtain sufficient stiffness to transmit force to the user because the soft fabric comprising the exosuit deforms when a force acts on the suit. Because of this limitation, it is challenging to deliver a strong force to the user. Even though the force is delivered, it is accompanied by latency because structural deformation of the suit occurs [16]. Moreover, because the suit deformation dissipates a portion of the energy needed to be delivered to the user [17], the force transfer efficiency is not higher than that of the exoskeleton, providing sufficient stiffness while delivering the force.

Several researchers have attempted to overcome these limitations of exosuits. Several studies have presented a design of exosuits to enhance structural stiffness. Wehner et al. attained the structural stiffness of an exosuit by introducing the concept of a key anchor, which supports the assistive force, and a virtual anchor, which redirects the force from the actuator to the key anchor through the surface path on the body [18]. However, this method has another limitation: it weakens the benefits of the exosuit by increasing its weight and design complexity because the connectors that link the virtual anchors are arranged along triangulation paths and not the direct linear paths, to transmit the force effectively in the sagittal plane. Asbeck et al. designed an ankle assistance exosuit with multiple webbings that can transmit force from the pelvis to the ankle in multiple directions to maximize the stiffness by minimizing suit displacement [19]. Although the stiffness of the exosuit could be maximally obtained as much as possible through this design, it requires anchor points other than the assisted ankle, such that the structure of the exosuit becomes complex. Additionally, a study for an attempt to characterize the exosuit system also exists. Quinlivan et al. outlined a methodology for characterizing the structured functional textiles of exosuits and evaluated several factors that lead to different human-suit series stiffness by using that methodology [20]. However, it has a limitation that the validation for the methodology was executed with only one human subject. In addition, the effect of crucial component of exosuits for transmitting the force, the anchor point, was not considered.

To advance the endeavors to overcome structural limitations of the exosuits, our group focuses on characterizing the force capability and human-suit stiffness corresponding anchor point positions for a conventional exosuit design. The force capability indicates the maximum value of the force transmitted through the exosuit and the human-suit stiffness indicates the series stiffness of human body and textiles of exosuits. First, we analyzed and identified the trend of the performance of the hip-assisting exosuit corresponding to changes in the anchor point position. The anchor points for transmitting the assistive force must exist on the exosuit. In the case of the exosuit for hip assist, the anchor points are at the waist belt and thigh brace. The anchor points on the waist belt are located at the back of the hip, and those on the thigh brace are located at the back of the thigh. The position of the anchor points is highly related to the capability of force transmission and the human-suit stiffness. Thus, we aimed to determine the correlation between the position of the anchor points and the capability of force transmission and human-suit stiffness.

We hypothesized that a greater distance between anchor points leads to a higher force capability owing to the wider permissible deformation range of the exosuit. However, we did not have a strong hypothesis regarding the trend of the human-suit stiffness corresponding to the anchoring points. A waist belt and thigh braces with three different anchor points positioned vertically were produced to verify the hypothesis and to characterize performance. By modifying the position of the anchor point, we searched for changes in the capability to transmit assistive force as the position changes. The assistive force transmission capability changed nonlinearly as the condition level of the anchor points changed. Furthermore, the human-suit stiffness model according to each anchor point position was obtained by measuring the cable position and transmitted force. The force capability and human-suit stiffness were changed in the opposite direction according to the variation of the anchor point position. Finally, we discussed the relationship between the anchor point position and the force capability, and the anchor point position and the human-suit stiffness, suggesting a design guide for exosuits based on this discussion.

## Method

### Review of hip exosuit

**Fig 1:**
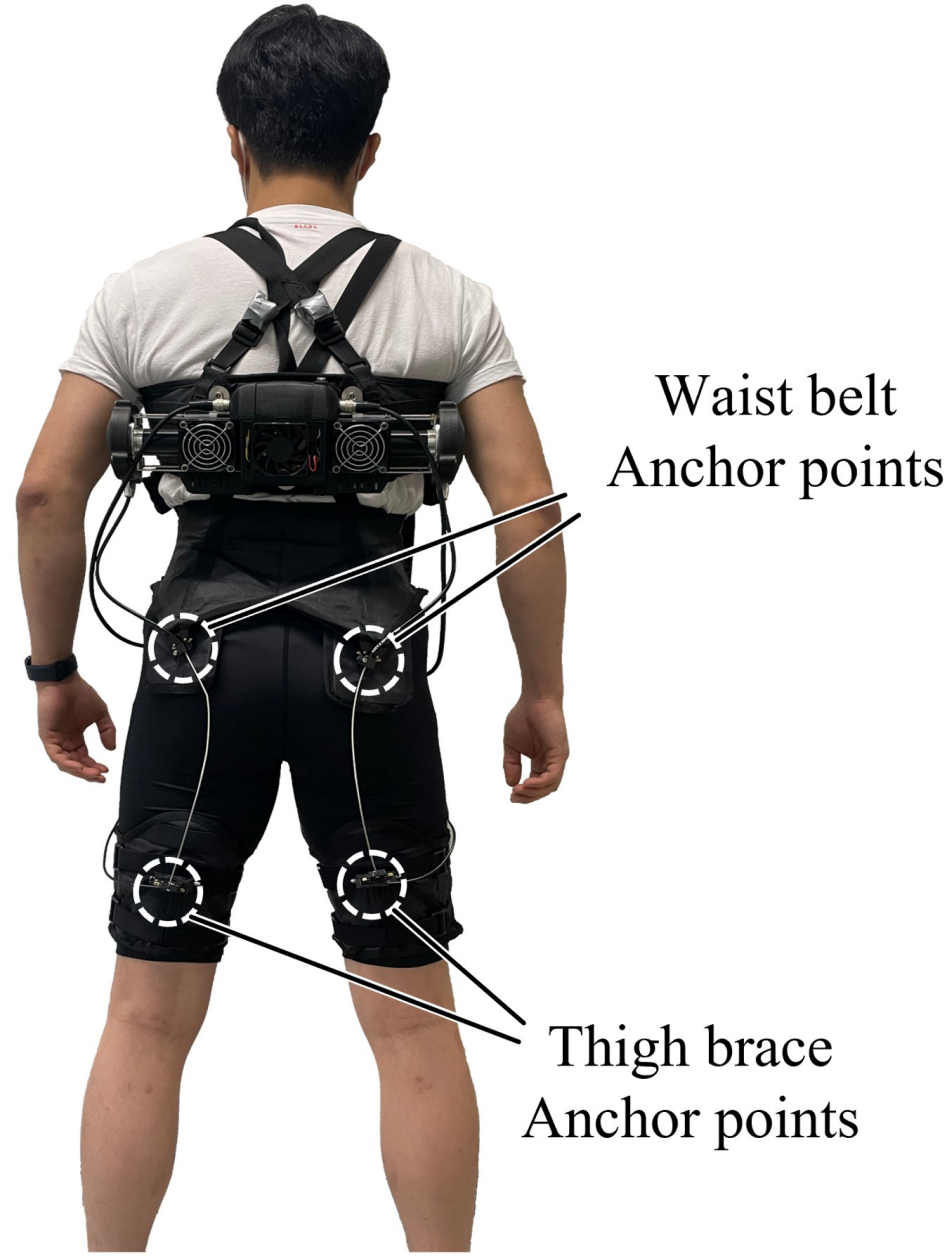
The overall shape of the exosuit and positions of the anchor points.

An exosuit comprises actuators that generate an assistive force, a suit made of textile that delivers the assistive force, and cables that transmit the force to the suit. The suit is divided into a waist belt and thigh braces, depending on the part where it is worn. To effectively transmit the assistive force generated from the actuator to the user, anchor points are required where the cable can be fastened to the exosuit comprising the textile. In the case of the exosuit that assists hip extension muscles, which we set as the target of analysis, four anchor points exist, with two points at the waist belt and two points at the thigh brace for each left and right. The conventional anchor points were designed based on the drawings presented in [21]. The overall shape of the exosuit and positions of the anchor points are shown in Fig 1.

### Experimental design

**Fig 2:**
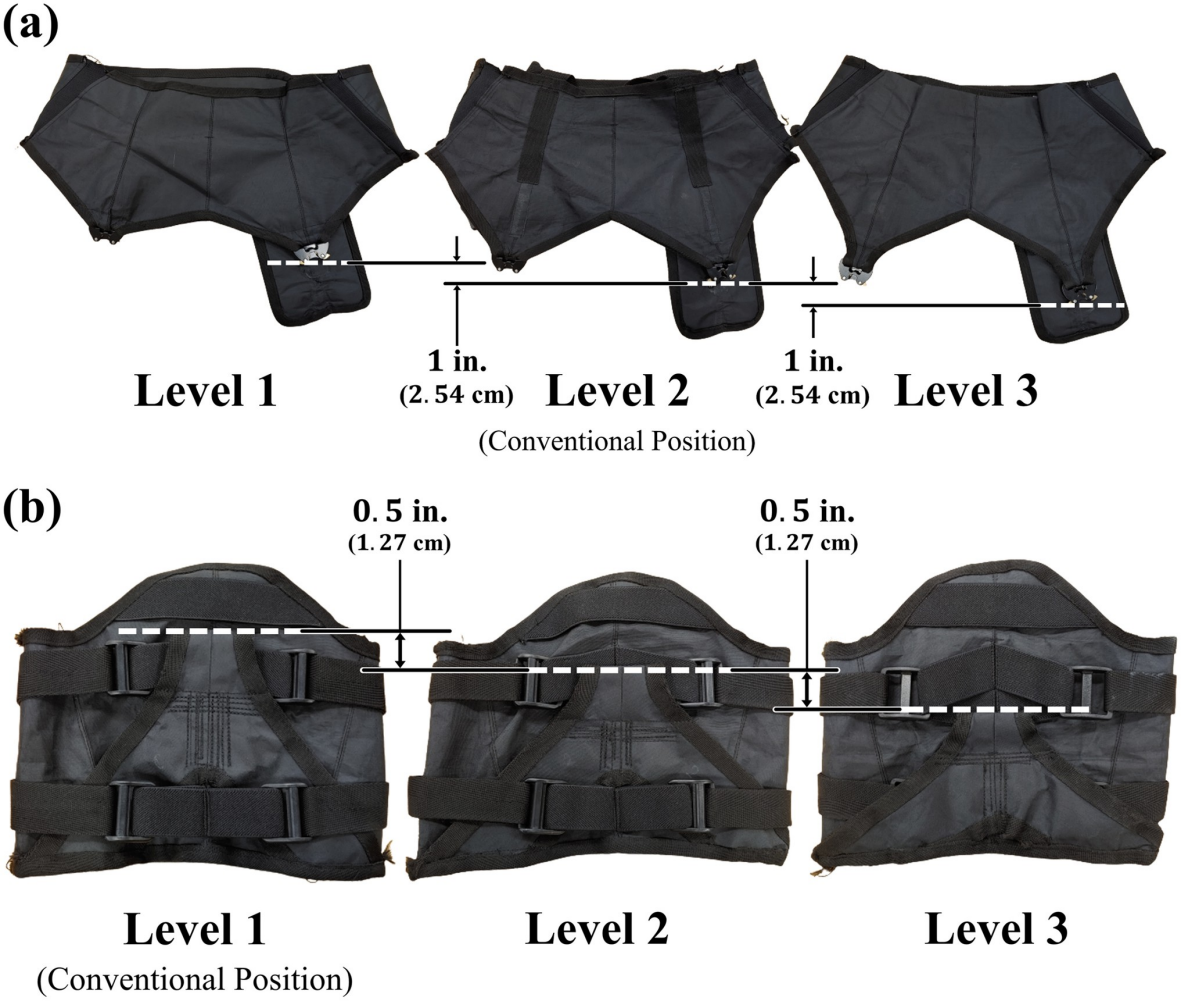
The conventional and new positions of the anchor points in each part of exosuit. (a) Anchor points of waist belt. (b) Anchor points of thigh brace; white dotted lines denote the positions of anchor points in each condition.

To determine the changes in the force capability and human-suit stiffness model corresponding to the anchor point positions, three different positions for the experiment were set at the waist belt and thigh braces. One of the three positions is identical to that of the conventional anchor point. The new anchor points for the waist belt were located 1 in (2.54 cm) from the conventional anchor points in the upward and downward directions. In contrast, the new anchor points for the thigh brace were located 0.5 in (1.27 cm) and 1 in (2.54 cm) from the conventional position in the downward direction, because no space for positioning the new anchor points was available in the upward direction. In addition, the anchor points for the thigh brace were separated by 0.5 in (1.27 cm) because the current design pattern for the thigh brace did not allow the new anchor points to be separated by 1 in (2.54 cm). The new anchor points of the waist belt and thigh brace were divided into three levels. The anchor point at the uppermost position was Level 1, that at the mid position was Level 2, and that at the undermost position was Level 3. Fig 2 shows the conventional and new positions of the anchor points.

Experiments were conducted under nine conditions resulting from the combination of three different anchor point conditions for the waist belt and thigh brace. The force capability and human-suit stiffness model were measured for eight subjects (25.88 ± 1.13 years old; 68.25 ± 6.94 kg; 171.25 ± 4.95 cm; mean ± std). To prevent alteration of the exosuit position depending on the subject, the position of the suit was regulated to align the upper end of the waist belt with the pelvis and to locate the lower end of the thigh brace 3 cm above the knee. Experiments under nine different conditions were repeatedly executed ten times each for subject. This study was approved by the Chung-ang University Institutional Review Board, and all procedures were conducted in accordance with the approved study protocol. All participants provided written informed consent before participation, and the nature and possible consequences of the study were explained to them.

**Fig 3:**
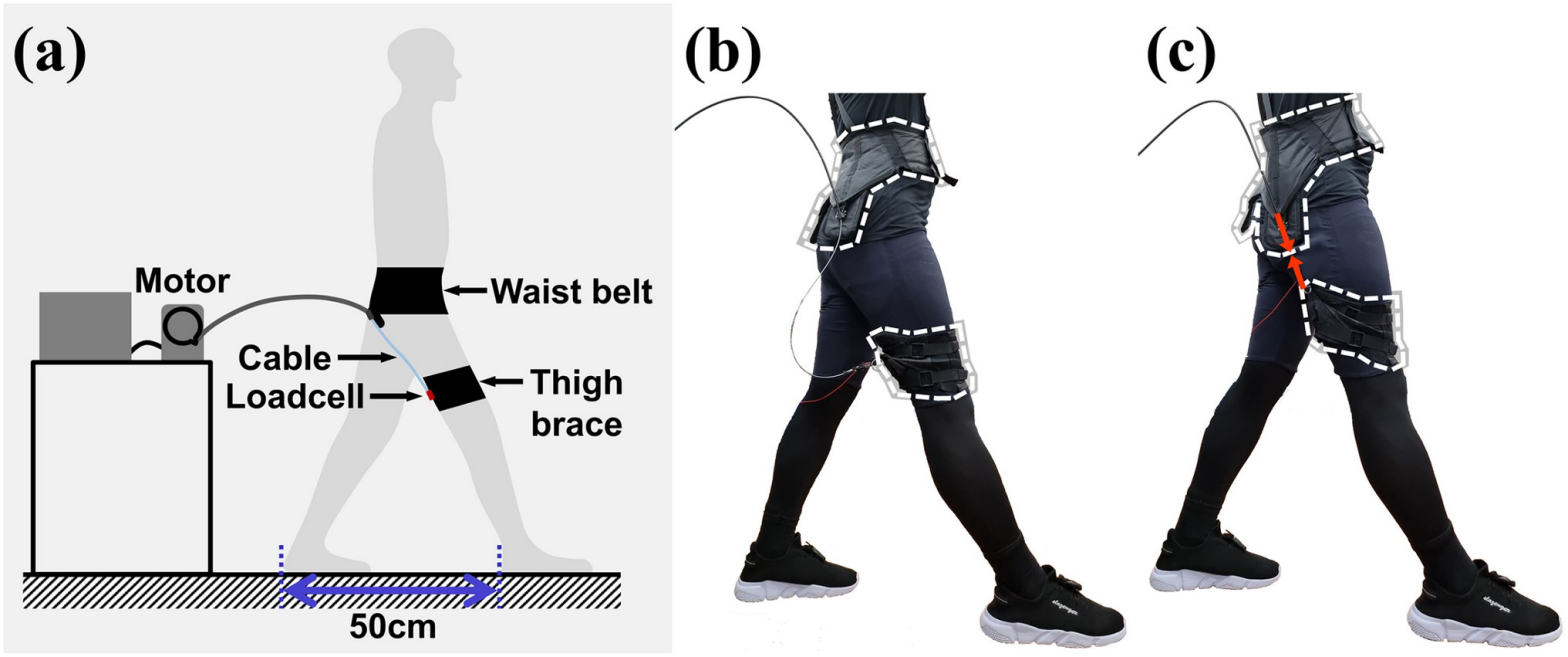
The procedure of the experiment. (a) Overview for configuration of the experiment. (b) The state when the cable was released during the experiment. (c) The state when the cable was pulled during the experiment; Areas surrounded by white dotted line denote the waist belt and thigh brace respectively, and red arrows denote the direction of the force.

The experimental procedure was identical for every condition as follows: The subjects wearing the exosuit on the designated position of the body were asked to assume a posture that spread their feet 50 cm apart. This posture was similar to that at 13% gait cycle, which is close to when assistive force was maximally applied based on previous studies [22, 23]. While the subject maintained posture, an actuator (RE 50, single-axis DC motor with 181:1 gear set, Maxon Corp., Sachseln, Switzerland) on the testbed pulled a Bowden cable (FCP-04DB, RESPONSE, Taichung, Taiwan, used with cable housing, BHL100, Jagwire, Changhua City, Taiwan), which was connected to the anchor points of the exosuit. A commercial motor driver (EPOS4, Maxon Corp., Sachseln, Switzerland) is used to control the actuator. The actuator pulled the cable until its remaining length reached 2 cm. The 2 cm of distance was a safety limit for avoiding collisions between the endpoint of the cable and the anchor point of the thigh brace. The cable was released to a point where no tension was applied to it after it reached the safety limit. A cycle, which entailed pulling and releasing the cable, was implemented ten times repeatedly. As the cycle was executed, the force transmitted to the subject was measured using a load cell (LSB205, Futek Advanced Sensor Technology Inc., CA, USA) that was fixed at the anchor point of the thigh brace. Fig 3 shows the process of the experimental cycle. To minimize the order effect of the anchor point conditions, the order of the experimental conditions was randomized with a Latin square matrix.

### Evaluation index

The force capability and human-suit stiffness model were measured to evaluate the effect of changes in the anchor point position. Force capability refers to the range of maximal assistive force that can be applied under the corresponding anchor point condition. Force capability can be represented by the peak force measured after the cable is pulled to the safety limit. For the evaluation index, the average value of the repeatedly measured peak force was used for each of the nine anchor point conditions. The force capability is expected to change according to the cable stroke, which differs in response to the combination of anchor points of the waist and thigh braces.

The human-suit stiffness model was obtained by fitting the measured data based on the stiffness model provided in [24]. The data for cable tension were measured through the load cell, and the travel length of the cable was measured by an encoder on the motor axis. The human-suit stiffness model was obtained by curve fitting all the data repeatedly measured ten times for each anchor point condition. The stiffness model is expressed as follows:

$$x=\;C_{1}*\text{ln}\left(C_{2}*F+1\right)$$ (1)

where ***x*** is the length of the pulled cable from the point where the force is measured by the load cell; ***F*** is the force measured by the load cell; and ***C***_1_, ***C***_2_ are the coefficients of the model. The effect of the coefficients in the equation is explained in the Discussion.

### Statistics

A statistical analysis was performed to analyze the effect of the anchor point condition change on the force capability and human-suit stiffness using MATLAB (R2021b, MathWorks, MA, USA). The mean of the force capability and the standard error of the mean for each of the nine conditions were used for the analysis of the force capability. The coefficients of the stiffness model were used to analyze the human-suit stiffness. The normality of the respective evaluation indices was tested for every anchor point condition using the Jarque–Bera test. Different analysis methods were selected depending on the test results. When normality was satisfied, a two-way ANOVA was adopted to test whether a significant difference was observed when the anchor point condition changed. If a significant difference existed, a post-hoc analysis was conducted using Tukey’s HSD test. In contrast, the Scheirer–Ray–Hare test was conducted to verify whether a significant difference was observed when normality was not satisfied. If a difference existed, a post-hoc analysis using the Dunn–Sidák method was executed. The significance level was set at 5% for all statistical analyses.

## Results

### Force capability

To perform statistical analyses, normality tests were conducted for force capability of each anchor point condition. Because all the data were satisfied the normality, the two-way ANOVA was conducted to identify whether a significant difference exists. The results of the two-way ANOVA revealed that the force capability had a significant difference for both changes in the waist belt and thigh brace (p < 0.001 for waist belt, p = 0.017 for thigh brace). To ascertain which experimental conditions had statistically significant differences, a post-hoc analysis was implemented. For the waist belt, differences were observed between Level 1 and 2 conditions and between Level 1 and 3 conditions. The force capabilities for Level 1, Level 2, and Level 3 conditions were measured as 316.4 ± 16.4 *N*, 240.6 ± 13.9 *N*, and 214.6 ± 12.5 *N*, respectively. Meanwhile, a statistically significant difference was observed only between Level 1 and 3 conditions for the thigh brace. Respective force capabilities were measured as 234.4 ± 14.3 *N*, 247.1 ± 15.2 *N*, and 290.2 ± 18.7 *N* for Level 1, Level 2, and Level 3 conditions, respectively.

**Fig 4:**
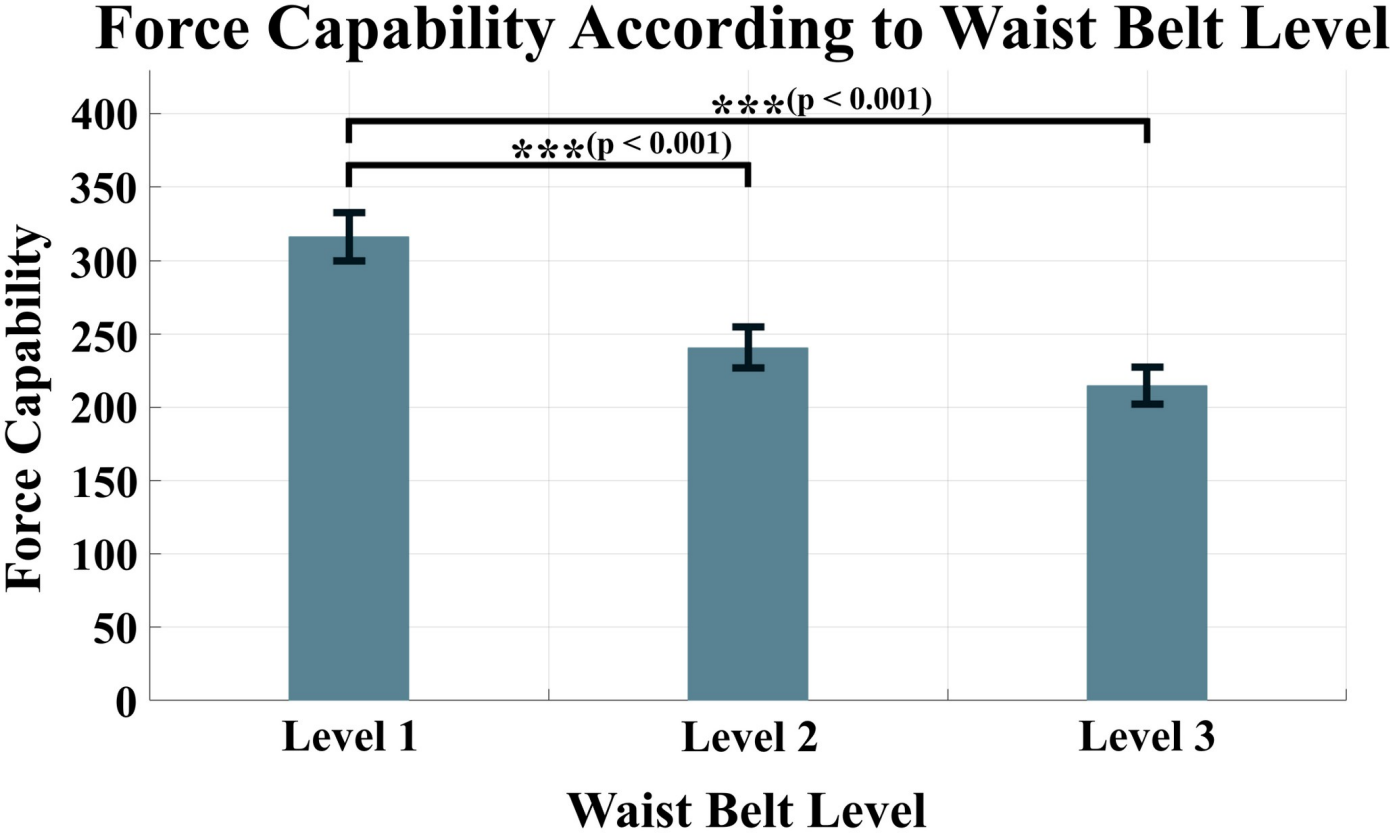
Force capability according to the condition levels of the waist belt. p < 0.001 between Level 1 and Level 2 conditions, and Level 1 and Level 3 conditions.

**Fig 5:**
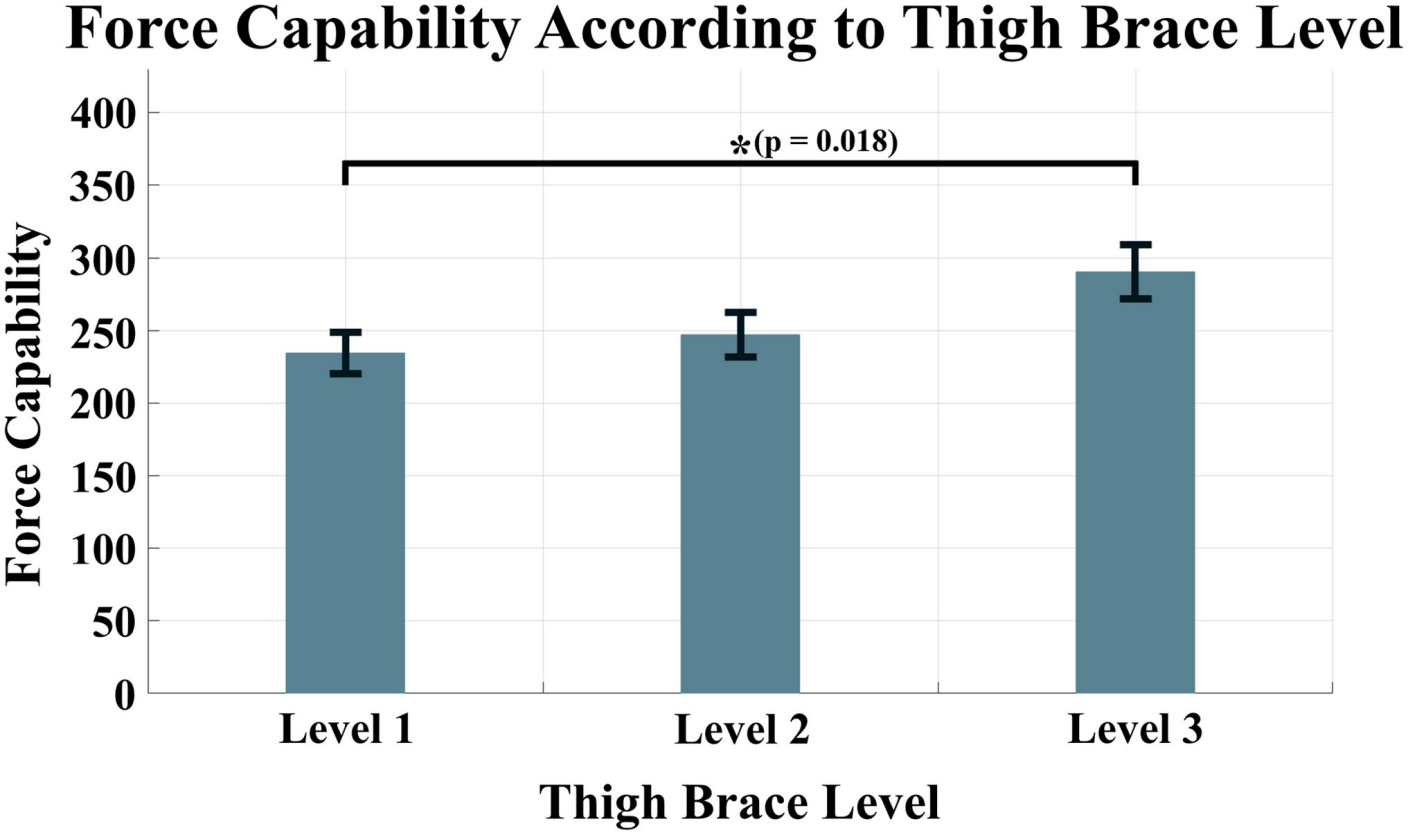
Force capability according to the condition levels of the thigh brace. p = 0.018 between Level 1 and Level 3 conditions.

Note that the force capability changes nonlinearly depending on the level of the anchor points at the waist belt and thigh braces. Figs 4 and 5 show the changing force capability according to the condition levels of the waist belt and thigh brace, respectively. Table 1 lists the average force capability of the eight subjects according to the conditions.

**Table 1:** Mean and standard error of the mean for force capability according to anchor point level change.

Force Capability [N] (Mean ± SEM)		Waist belt level			
		1	2	3	Total
**Thigh brace level**	**1**	292.6 ± 22.5	219.4 ± 18.2	191.1 ± 20.2	234.4 ± 14.3
	**2**	300.2 ± 27.4	234.8 ± 22.8	206.2 ± 18.6	247.1 ± 15.2
	**3**	356.3 ± 32.3	267.7 ± 29.8	246.6 ± 23.2	290.2 ± 18.7
	**Total**	316.4 ± 16.4	240.6 ± 13.9	214.6 ± 12.5	

### Human-suit stiffness model

**Fig 6:**
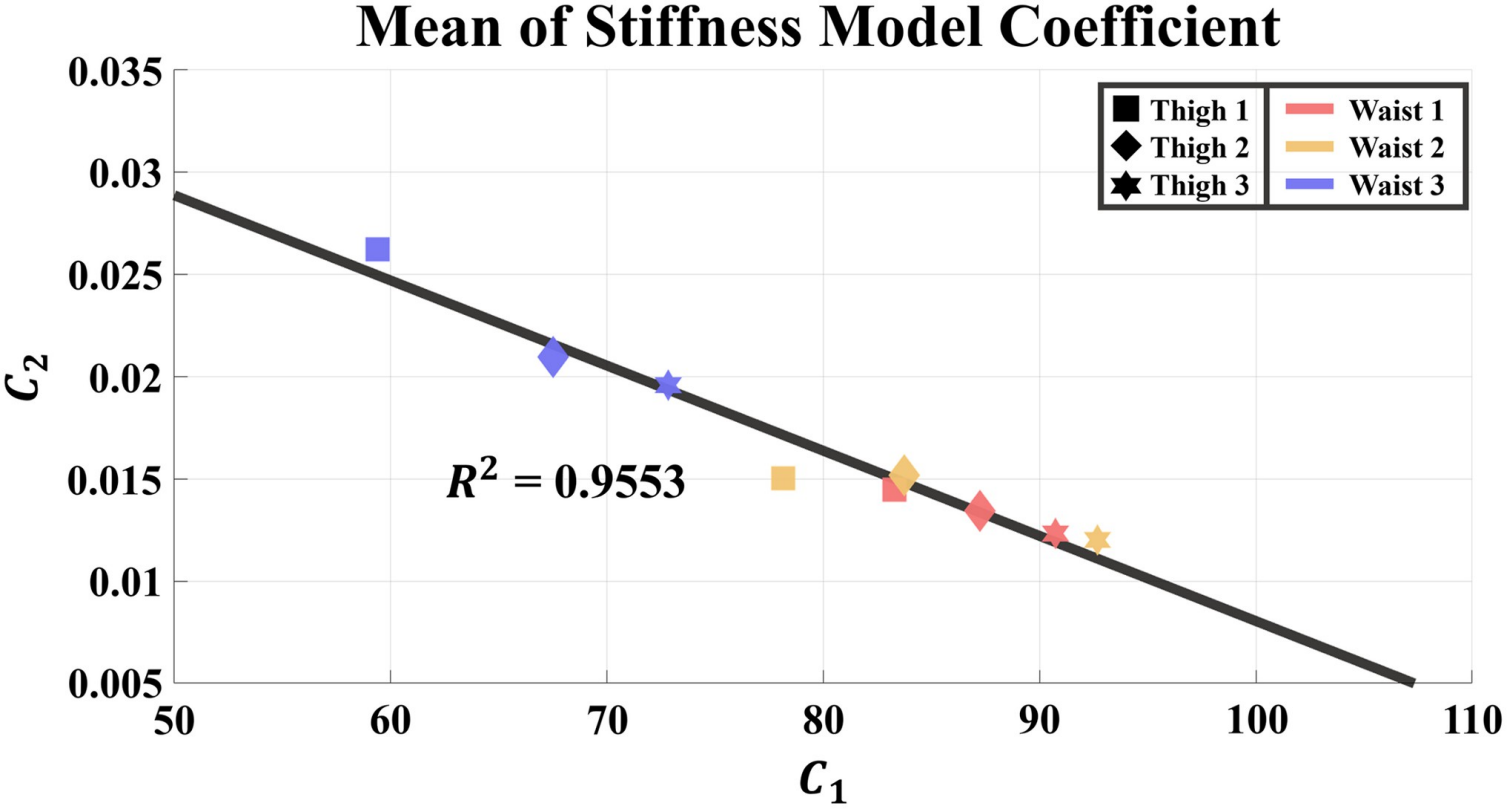
The coefficients of the stiffness model according to condition levels of anchor points and a trend line of the coefficients. Each point denotes the mean coefficient of eight subjects in each condition.

To analyze the obtained stiffness model, the change in the stiffness model coefficients according to the anchor point conditions was identified. The coefficients of the stiffness model for each subject are listed in S1 Table. The changing trend of the stiffness coefficients can be verified from the averaged values of the overall subjects for each anchor point condition. Fig 6 presents the mean values of the stiffness coefficients for all subjects and their trend line. As the condition level of the anchor point at the waist belt changed from Level 1 to Level 3, coefficient ***C***_1_ decreased, whereas coefficient ***C***_2_ increased. In contrast, coefficient ***C***_1_ increased while coefficient ***C***_2_ decreased as the condition level of the anchor point at the thigh brace changed from Level 1 to Level 3.

Normality tests were conducted to perform statistical analyses for the two coefficients. The ***C***_1_ coefficient satisfied the normality for every condition, while the ***C***_2_ coefficient did not satisfy the normality for a combination in which the waist belt anchor point and thigh brace anchor point had condition levels of three and one, respectively. A two-way ANOVA was implemented for the coefficient ***C***_1_ because it was normally distributed for every condition. Therefore, a significant difference was identified for the conditions of the anchor point at the waist belt and the thigh brace (p < 0.001 for waist belt level and thigh brace level). The Tukey HSD test was used to confirm which conditions had significant differences between each other. In the case of the waist belt, differences were shown between condition Level 1 and condition Level 3 and between condition Level 2 and condition Level 3. Meanwhile, for the thigh brace, differences were shown between condition Level 1 and condition Level 2, and between condition Level 1 and condition Level 3. In contrast, the Scheirer–Ray–Hare test was executed for coefficient ***C***_2_ because it did not satisfy normality. Statistically significant differences were observed only for the change in the waist belt anchor point (p < 0.001 for waist belt level, p = 0.141 for thigh brace level). The differences were shown between condition Level 1 and condition Level 3, and condition Level 2 and condition Level 3, as a result of the post-hoc analysis.

Owing to the nonlinearity of the stiffness model, the coefficients of the model represent only this tendency. Hence, for a more practical analysis, we divided the section according to the magnitude of the force. The upper limit of the force for the analysis was set to 200 N, considering that the minimum force capability of the subjects was 190 N. The entire range was divided into every 50 N, such that it was divided into four sections. The average stiffness of each section was calculated using the following equation:

$$k_{Avg}=\;\frac{F_{e}-\;F_{i}}{x_{e}-\;x_{i}}$$ (2)

where ***k***_Avg_ is the average stiffness of the range, which is divided according to the magnitude of the force; ***F***_e_ and ***F***_i_ are the values of the measured force at the endpoint and the initial point of the range, respectively; and ***x***_e_ and ***x***_i_ are the traveled length of the cable at the endpoint and the initial point of the range, respectively.

**Fig 7:**
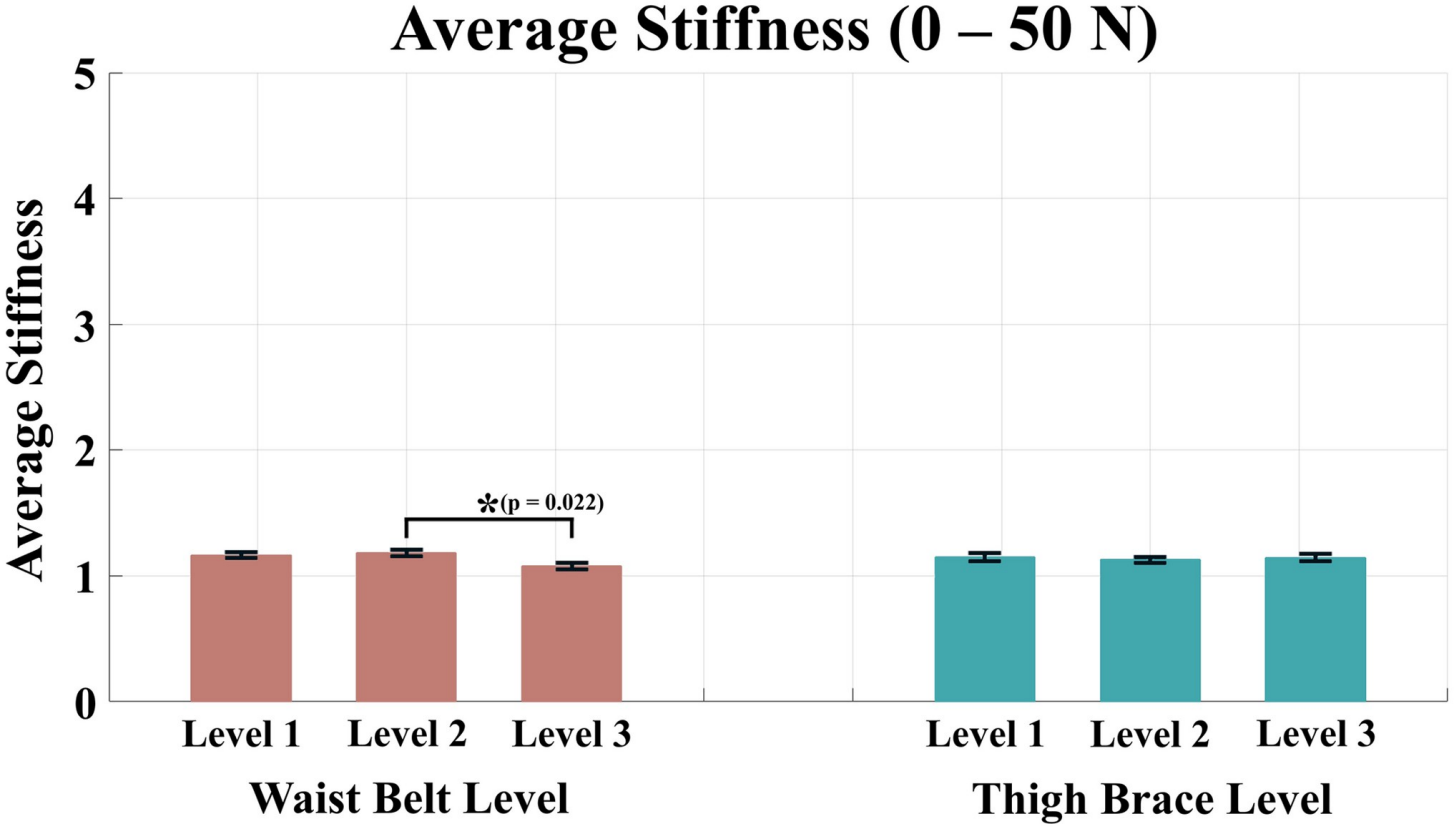
Average stiffness for the force range of 0–50 N. The red bars represent the mean value and the standard error of mean according to the waist belt level, and the green bars represent the mean value and the standard error of mean according to the thigh brace level.

**Fig 8:**
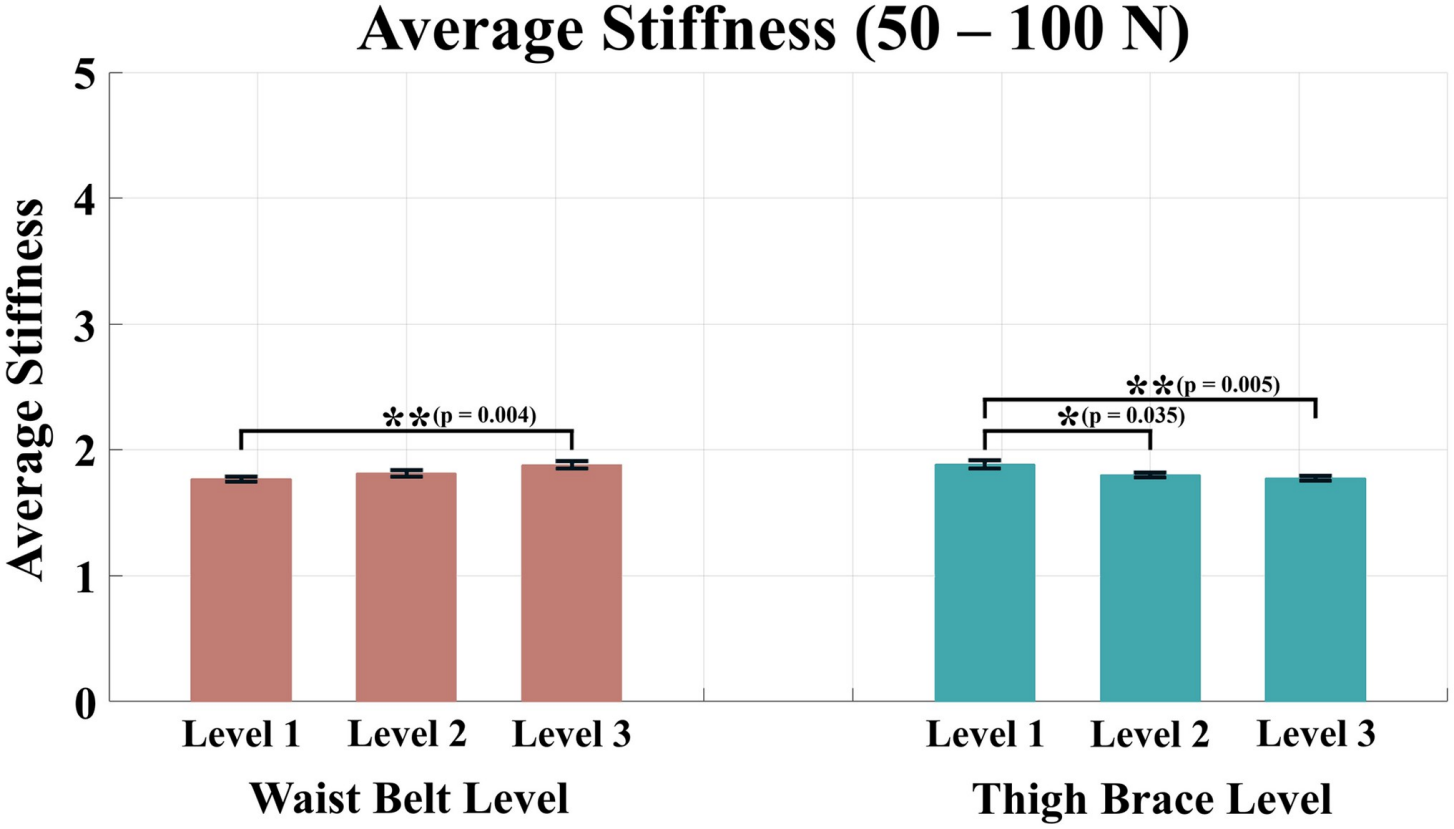
Average stiffness for the force range of 50–100 N. The red bars represent the mean value and the standard error of mean according to the waist belt level, and the green bars represent the mean value and the standard error of mean according to the thigh brace level.

**Fig 9:**
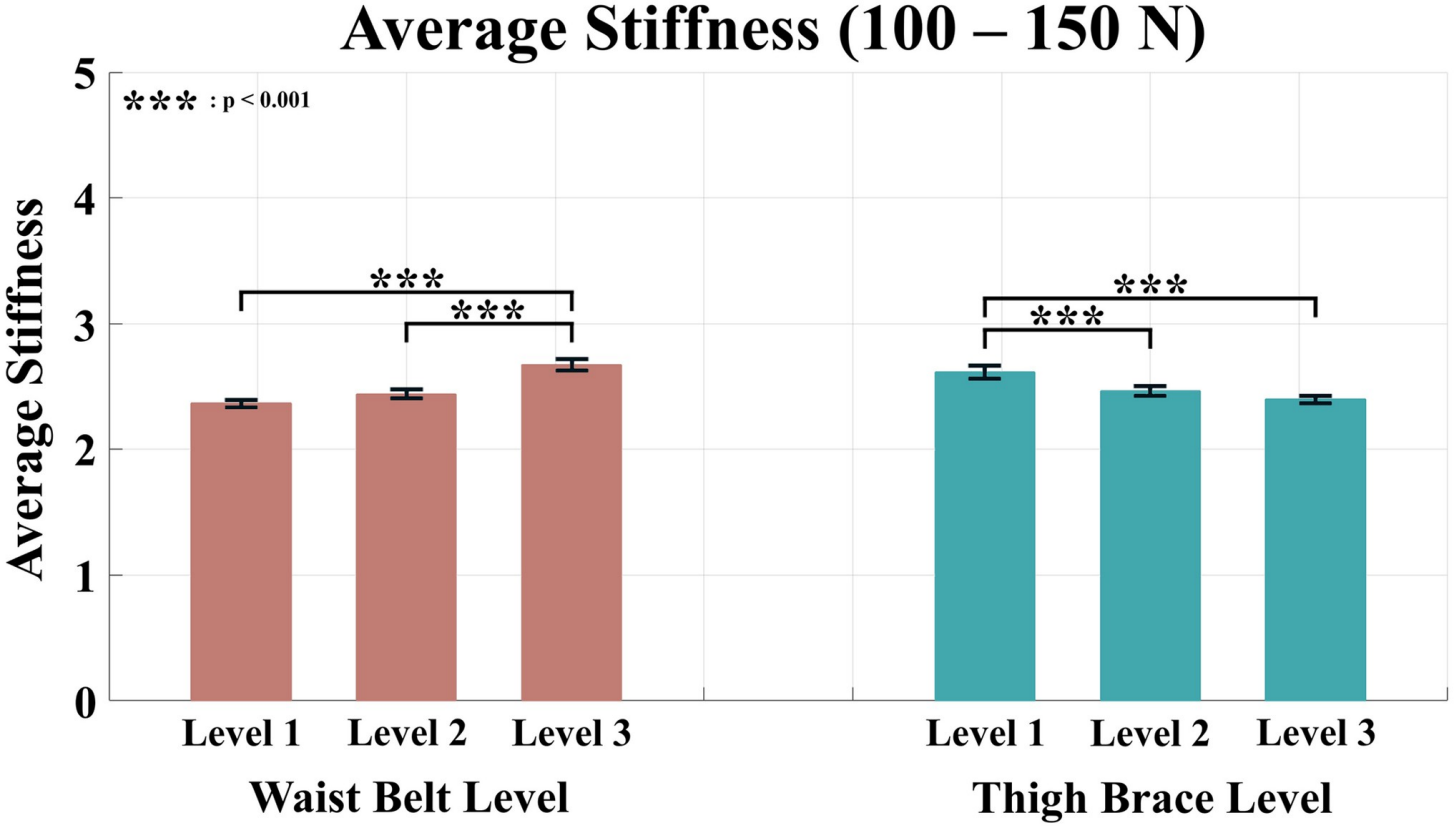
Average stiffness for the force range of 100–150 N. The red bars represent the mean value and the standard error of mean according to the waist belt level, and the green bars represent the mean value and the standard error of mean according to the thigh brace level.

**Fig 10:**
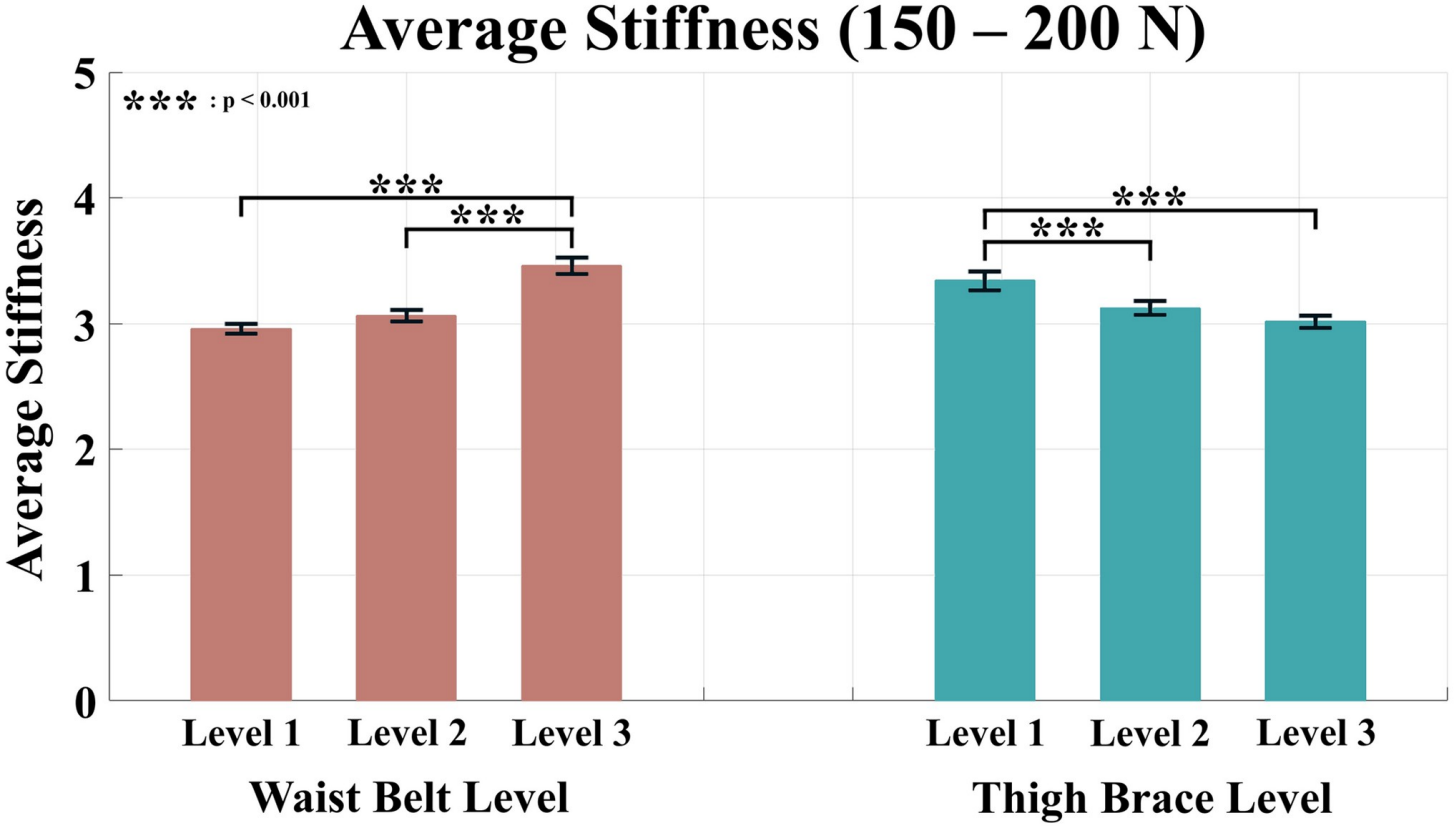
Average stiffness for the force range of 150–200 N. The red bars represent the mean value and the standard error of mean according to the waist belt level, and the green bars represent the mean value and the standard error of mean according to the thigh brace level.

Tables 2–5 list the average stiffness of each section according to the anchor point conditions. The red color in the Tables 2–5 denotes the maximum average stiffness of each subject, and the blue color denotes the minimum average stiffness of each subject. For the condition levels of the waist belt and thigh brace, a two-way ANOVA was performed because all the values of averaged stiffness satisfied the normality. As a result of the analysis, all sections except the range of 0–50 N showed significant differences in the condition level of the waist belt and thigh brace. The results of the analysis for the average stiffness of each force range are shown in Figs 7–10.

**Table 2:** Average stiffness for the force range of 0–50 N. The red and blue colors denote the maximum and minimum values for each subject, respectively.

Average Stiffness (0–50 N)																									
Anchor point level		Waist																							
		1	2	3	1	2	3	1	2	3	1	2	3	1	2	3	1	2	3	1	2	3	1	2	3
**Thigh**	**1**	1.24	1.34	0.95	1.27	1.29	1.11	1.05	1.07	1.15	1.23	1.33	1.32	1.33	1.25	1.12	1.04	1.00	0.83	1.23	1.36	1.19	0.90	1.00	0.95
	**2**	1.11	1.10	1.04	1.22	1.15	1.02	1.14	1.11	1.14	1.27	1.23	1.26	1.17	1.25	1.22	1.10	0.96	0.84	1.24	1.28	1.12	1.01	1.04	1.03
	**3**	1.12	1.29	1.09	1.16	1.17	1.05	1.24	1.16	1.13	1.31	1.39	1.23	1.25	1.34	1.13	1.09	1.01	0.82	1.27	1.18	1.10	0.98	0.99	1.00
**Subject**		**1**			**2**			**3**			**4**			**5**			**6**			**7**			**8**		

**Table 3:** Average stiffness for the force range of 50–100 N. The red and blue colors denote the maximum and minimum values for each subject, respectively.

Average Stiffness (50–100 N)																									
Anchor point level		Waist																							
		1	2	3	1	2	3	1	2	3	1	2	3	1	2	3	1	2	3	1	2	3	1	2	3
**Thigh**	**1**	1.81	1.96	1.82	1.83	1.98	2.05	1.75	1.78	1.86	1.93	2.01	2.16	1.94	1.96	2.03	1.56	1.69	1.89	1.90	2.03	2.23	1.61	1.74	1.75
	**2**	1.74	1.81	1.85	1.83	1.83	1.87	1.80	1.81	1.81	1.78	1.71	1.89	1.74	1.82	1.98	1.61	1.57	1.74	1.84	1.94	2.01	1.74	1.66	1.79
	**3**	1.79	1.87	1.77	1.72	1.82	1.81	1.83	1.77	1.79	1.80	1.88	1.84	1.77	1.83	1.78	1.61	1.54	1.68	1.87	1.89	1.92	1.61	1.58	1.80
**Subject**		**1**			**2**			**3**			**4**			**5**			**6**			**7**			**8**		

**Table 4:** Average stiffness for the force range of 100–150 N. The red and blue colors denote the maximum and minimum values for each subject, respectively.

Average Stiffness (100–150 N)																									
Anchor point level		Waist																							
		1	2	3	1	2	3	1	2	3	1	2	3	1	2	3	1	2	3	1	2	3	1	2	3
**Thigh**	**1**	2.39	2.58	2.68	2.40	2.66	2.99	2.44	2.48	2.56	2.62	2.69	3.00	2.55	2.66	2.92	2.08	2.36	2.90	2.55	2.70	3.24	2.30	2.47	2.54
	**2**	2.36	2.50	2.65	2.42	2.50	2.71	2.46	2.50	2.48	2.29	2.18	2.53	2.30	2.39	2.73	2.13	2.17	2.60	2.44	2.60	2.88	2.45	2.28	2.54
	**3**	2.44	2.44	2.46	2.27	2.46	2.55	2.41	2.37	2.44	2.29	2.37	2.45	2.30	2.32	2.42	2.13	2.07	2.52	2.47	2.60	2.74	2.24	2.17	2.58
**Subject**		**1**			**2**			**3**			**4**			**5**			**6**			**7**			**8**		

**Table 5:** Average stiffness for the force range of 150–200 N. The red and blue colors denote the maximum and minimum values for each subject, respectively.

Average Stiffness (150–200N)																									
Anchor point level		Waist																							
		1	2	3	1	2	3	1	2	3	1	2	3	1	2	3	1	2	3	1	2	3	1	2	3
**Thigh**	**1**	2.96	3.19	3.53	2.96	3.33	3.91	3.13	3.17	3.27	3.31	3.37	3.83	3.16	3.36	3.81	2.59	3.03	3.91	3.21	3.36	4.26	2.99	3.19	3.33
	**2**	2.98	3.20	3.44	3.02	3.17	3.54	3.11	3.19	3.14	2.80	2.66	3.15	2.87	2.96	3.47	2.64	2.77	3.46	3.05	3.25	3.74	3.16	2.90	3.28
	**3**	3.09	3.02	3.13	2.83	3.11	3.29	2.99	2.97	3.09	2.79	2.86	3.06	2.83	2.80	3.06	2.64	2.59	3.35	3.06	3.30	3.55	2.86	2.76	3.36
**Subject**		**1**			**2**			**3**			**4**			**5**			**6**			**7**			**8**		

For the range of 0–50 N, a significant difference was revealed only for the waist belt level (p = 0.017 for the waist belt level, p = 0.851 for the thigh brace level). The difference was identified between condition Levels 2 and 3 as a result of the post-hoc analysis.

In contrast, for the range of 50–100 N, significant differences were shown for both the waist belt level and thigh brace level (p = 0.006 for waist belt level, p = 0.004 for thigh brace level). In the case of the waist belt, condition Levels 1 and 3 showed a difference. For the thigh brace, differences existed between condition Levels 1 and 2, and Levels 1 and 3.

The range of 100–150 N also showed significant differences in the level change of both waist belt and thigh brace conditions (p < 0.001 for waist belt level and thigh brace level). For the waist belt, significant differences were observed between condition Levels 1 and 3, and Levels 2 and 3. For the thigh brace, differences were observed between condition Levels 1 and 2, and Levels 1 and 3.

Similarly, the average stiffness was significantly different according to the condition level of both the waist belt and thigh braces in the range of 150–200 N (p < 0.001 for the waist belt level and thigh brace level). The result of the post-hoc analysis was identical to that of the 100–150 N range. Significant differences were observed between condition Levels 1 and 3 and Levels 2 and 3 of the waist belt. In the case of the thigh brace, differences were revealed between condition Levels 1 and 2 and Levels 1 and 3.

Overall, except for the range of 0–50 N, the stiffness increased when the condition level of the waist belt was higher and that of the thigh brace was lower. In other words, the closer the two anchor points of the waist belt and thigh brace, the higher the human-suit stiffness.

## Discussion

This study aimed to explore the characteristics of the force transmitted to the user of a soft-type wearable robot and the stiffness that changes according to the position of the anchor points of the robot system. To investigate the effect of the anchor point, we observed the change in the maximum assistive force capability and human-suit stiffness according to the anchor point positions.

### Force capability

The force capability showed a high correlation with the cable stroke. The greater the length of the cable stroke, the greater is the force capability. The maximum value of the force capability was shown when the condition levels of the waist belt and thigh brace were 1 and 3, respectively, for all the subjects.

**Fig 11:**
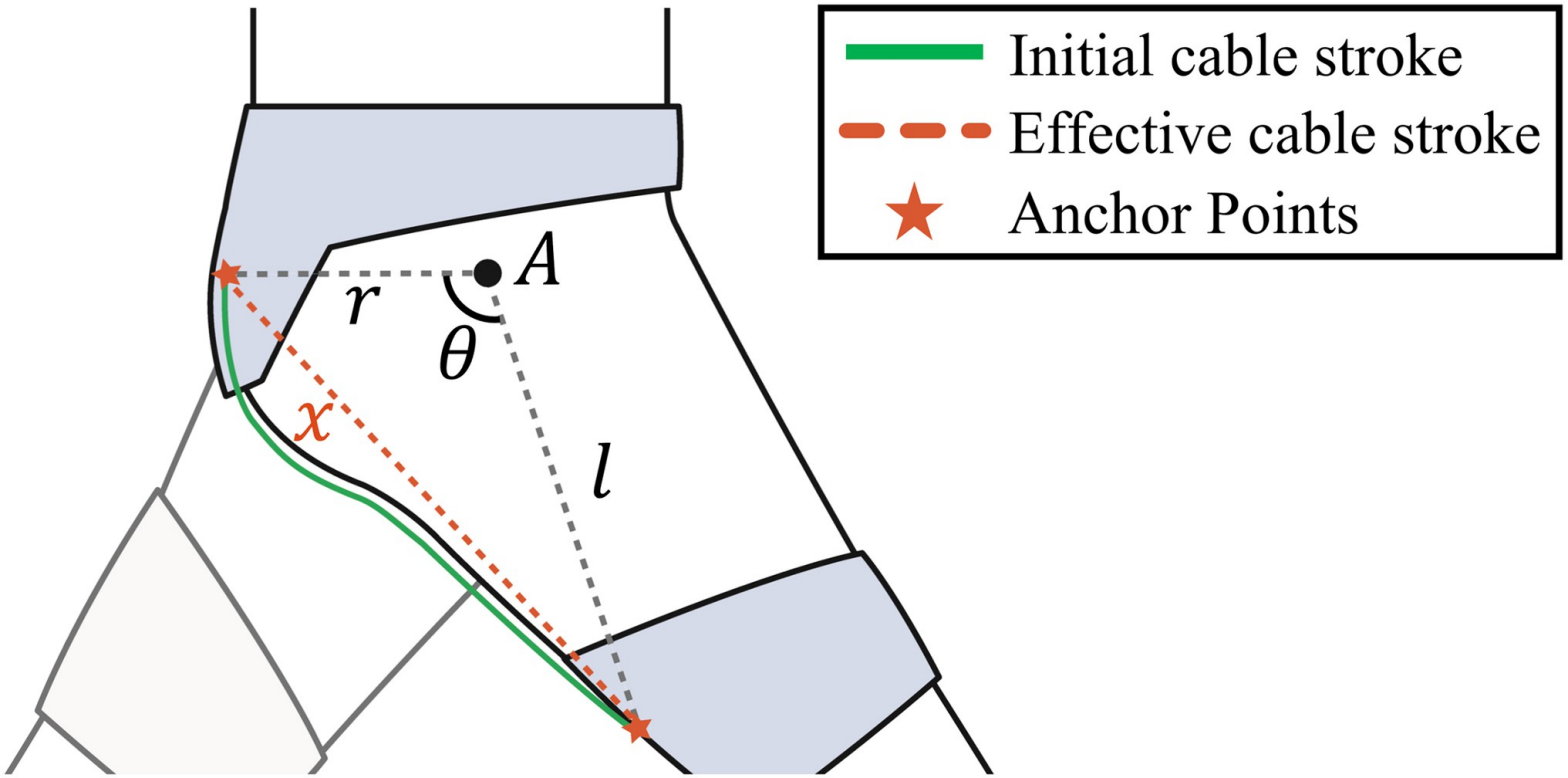
A simple schematic of the cable stroke length. A green line represents initial cable stroke along the body curve and a red dotted line represents effective cable stroke.

Meanwhile, the average force capability for all subjects changed nonlinearly, as the level of the anchor points at the waist belt and thigh braces changed. This is because the effective cable stroke changed nonlinearly owing to the body curve when the positions of the anchor points were changed. Fig 11 shows a simple model of the soft exosuit system, including anchor points and the human body.

The anchor point position of the waist belt and thigh brace changed by 1 in (2.54 cm) and 0.5 in (1.27 cm), respectively, as the condition levels were changed. Because the cable can deform along the surface curvature of the soft exosuit, the initial cable stroke differs linearly, depending on the anchor point level. However, when cable tension was applied while delivering assistive force to the wearer, the effective cable path became a straight line connecting the anchor points of the waist belt and the thigh brace because the exosuit and the human body easily deformed under the force.

In this situation, the effective cable stroke can be approximated as the direct distance between two anchor points, which is indicated as ***x*** in Fig 11, and the distance is calculated using the following equation, according to the law of cosine:

$$x=\;\sqrt{r^{2}+l^{2}-2rl\;\text{cos}\;\theta }$$ (3)

where point A is the center of rotation for the hip joint; ***θ*** is the angle between two connecting lines that connect point A and the anchor point of the waist belt, and point A and the anchor point of the thigh brace; ***r*** is the distance between point A and the anchor point of the waist belt; and ***l*** is the distance between point A and the anchor point of the thigh brace.

In this equation, the value of ***r*** is constant, and the values of ***l*** and ***θ*** change nonlinearly according to the levels of the anchor points of the waist belt and thigh brace. This shows that the effective cable path ***x***, which indicates the distance between the anchor points, changes nonlinearly as the anchor point positions change. Consequently, the force capability exhibits a nonlinear change with the change in the anchor point position.

In addition, the nonlinear characteristic of the force capability can be affected by the nonlinear stiffness of the human-exosuit system. The magnitude of the transmitted force is decided by the travel length of the cable and the stiffness of the system. Therefore, even if the cable stroke is identical, the force transmitted to the human body appears nonlinearly owing to the nonlinear stiffness. However, the relationship between the stiffness and the force capability could not be revealed through the experiment results. The experiment to obtain the data of force capability under the same condition of the cable stroke and different stiffness was not conducted. Further exploration of the relationship between the stiffness and the force capability would be required.

### Human-suit stiffness

Based on the analysis of the average stiffness in four divided sections according to the magnitude of the force, the closer the two anchor points of the waist belt and thigh brace, the higher the human-suit stiffness, which is in contrast to the effects on the force capability. We investigated the reasons for this trend as follows:

The stiffness of the human-suit system is calculated by the force that is measured from the load cell and the cable travel length, which is measured from the encoder at the actuator. Consequently, the stiffness is affected by the cable travel length and force transmitted from the actuator to the user. Each factor changes depending on the positions of the anchor points located at the waist belt and the thigh brace.

The force measured by the load cell is related to the degree to which the human body and suit intervene between the two anchor points at the waist belt and thigh brace. A partial loss of force generated by the actuator is induced by the interference of the body during the force-transmitting process from the actuator to the user. The majority of the loss is caused by friction between the cable and human-suit system. Therefore, the lost force increases when the contact between the cable and the user body or suit with the intervention of the human-suit system in the middle of the two anchor points.

**Fig 12:**
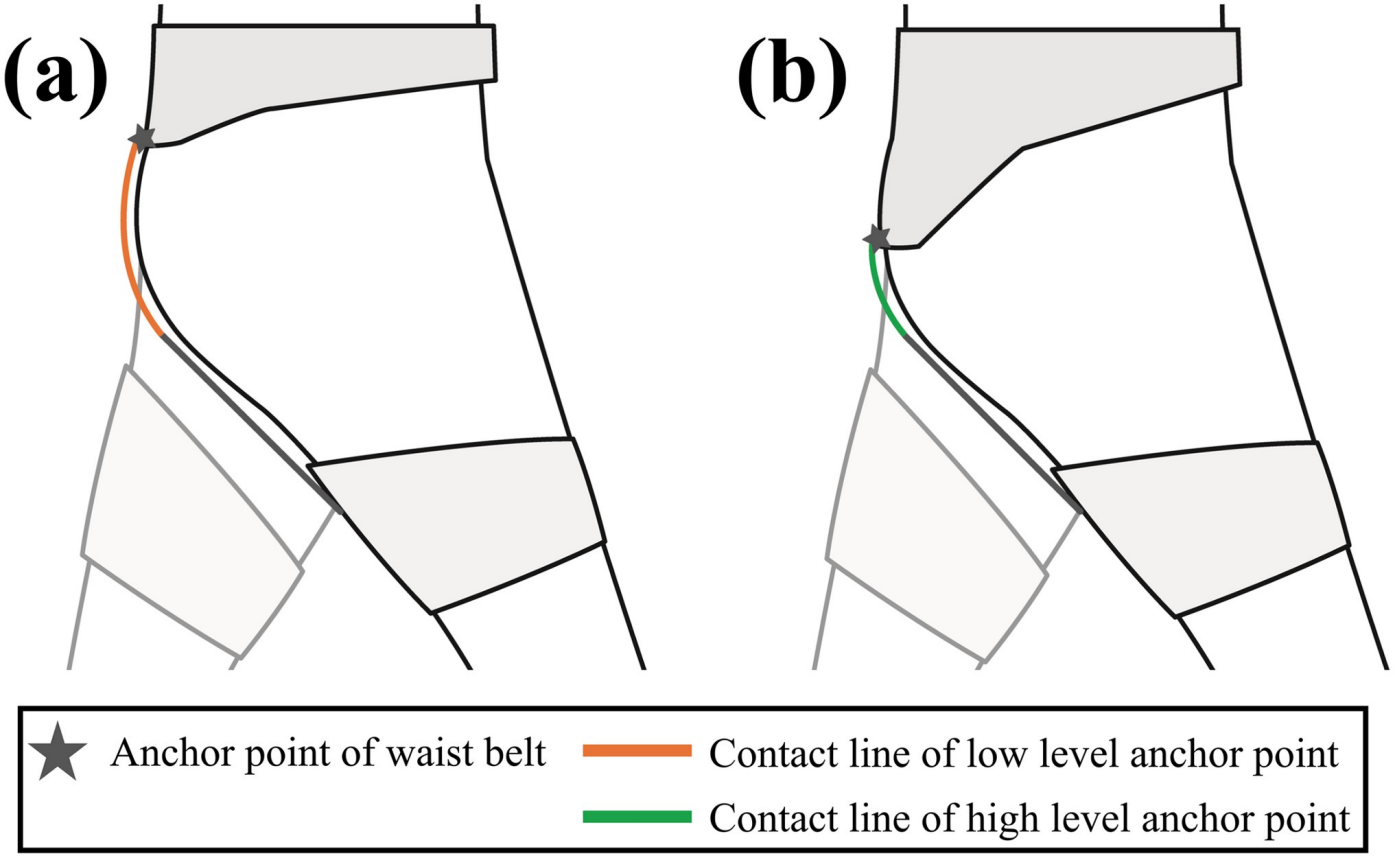
A simple schematic for contact line of the cable with the body. (a) The case when the anchor point level of the waist belt is low (Level 1). (b) The case when the anchor point level of the waist belt is high (Level 3). The length of the contact line is longer when the anchor point level of the waist belt is low.

Accordingly, the force measured by the load cell decreases because a smaller force is transmitted to the user for the same travel length of the cable. In other words, a smaller applied force for an identical travel length of the cable implies that the stiffness decreases. In summary, the stiffness is measured to be higher for the anchor point level that can avoid the interference of the human-suit system between the anchor points of the waist belt and thigh brace. Fig 12 shows a simple schematic of the cases in which the contact between the cable and body is relatively small and relatively large.

Additionally, the relationship between the position of the anchor points and the human-suit stiffness model coefficients can be observed despite of the nonlinearity of the stiffness model. The coefficient ***C***_1_ decreased as the condition level of the anchor point at the waist belt changed from Level 1 to Level 3 and the condition level of the anchor point at the thigh brace changed from Level 3 to Level 1. In contrast, the coefficient ***C***_2_ increased as the condition level of the anchor point at the waist belt changed from Level 1 to Level 3 and the condition level of the anchor point at the thigh brace changed from Level 3 to Level 1. To summarize, the two coefficients ***C***_1_ and ***C***_2_ changed in the opposite direction for an identical condition level change of the anchor points. According to Eq (1), smaller ***C***_1_ and ***C***_2_ values represent a higher stiffness because the travel length of the cable becomes shorter for the same magnitude of the force. Furthermore, ***C***_1_ has a larger impact on the travel length of the cable than ***C***_2_ because the value of ***C***_2_ is reduced by the logarithm. Consequently, a change in the coefficients of the human-suit stiffness model indicates that the stiffness increases as the anchor point of the waist belt and the anchor point of the thigh brace become closer.

### Example for using the characteristic for designing exosuit

We propose a guide for exosuit design by exploiting the characteristics of the force capability and human-suit stiffness that were identified through the experiments as follows. First, the cable stroke should be considered prior to obtaining a sufficient force capability to generate the torque required for assistance. In other words, designing the anchor point position to include the required force within the force-capability range is recommended. For example, an exosuit used to strengthen the body function should have a sufficient force-capability range [9]. Note that the force capability changes nonlinearly according to the change in anchor point level.

In the case of the exosuit, which aims to transmit low force effectively and not to obtain a high assistive force, designing the anchor point to increase stiffness is more appropriate than maximizing the force capability. In this manner, the exosuit can deliver the target assistive force to the wearer more effectively while minimizing loss. For example, an exosuit used for the rehabilitation of patient or that used to assist elderly people in performing physical activities needs to transmit a small assistive force effectively [25, 26]. The anchor points of the waist belt and thigh brace should be designed to maximize the stiffness within the range in which the target linear force can be generated. Note that the directions of the anchor point change when increasing the stiffness (Level 1 to Level 3 for the waist belt and Level 3 to Level 1 for the thigh brace) and expanding the force capability (Level 3 to Level 1 for the waist belt and Level 1 to Level 3 for the thigh brace) are opposite.

If the anchor points on the waist belt and thigh brace of the exosuit must have a different position than the explored ones in this study, the trend of the stiffness model coefficients derived from the experiment in this study can be used. The stiffness model coefficients ***C***_1_, ***C***_2_ exhibit a linear correlation as the anchor point levels change. Through this relationship, the new coefficients for the stiffness model with new anchor point positions can be estimated for the new exosuit design.

## Conclusion

This study aims to determine the effect of the anchor point position of soft wearable robots and provide design criteria for setting proper anchor points. First, we experimentally identified the change in force capability and human-suit stiffness model depending on the anchor point position. The force capability differs when the anchor point position changes because the cable stroke changes according to the anchor point positions. In addition, the shape of the human-suit stiffness model is affected by the anchor point position because the degree of user body interference changes with the anchor point. The coefficients of the human-suit stiffness model showed a tendency depending on the anchor point condition. Understanding the nature of components, such as soft textiles or the human body, would provide more insight into the deformable characteristics of soft wearable robots. For example, the information on force capability and human-suit stiffness that we provided can be used when other groups make an exosuit simulator or design a new exosuit.

Although the changing trend of the force capability and the human-suit stiffness model according to the anchor point position level were identified, this study is limited in that the degree of the condition change in the waist belt and thigh brace were not treated identically because of the practical issue of designing the proper suit pattern for the anchoring points. Owing to these non-uniform condition changes, the factors that are more effective could not be determined. In future work, we need to further explore the characteristics of the anchoring points, such as which is the major factor in changing the system characteristics, such as the force capability or human-suit stiffness model.

## Supporting information

S1 Table Coefficients of the stiffness model for subject 1 to subject 8. (PDF)
