# Supplementary material for: Characterizing force capability and stiffness of hip exosuits under different anchor points
Source: PLoS One. 2022 Aug 4;17(8):e0271764. doi: 10.1371/journal.pone.0271764 (PMC9352082; doi:10.1371/journal.pone.0271764)
Supplement: S1 Table — (PDF) [file pone.0271764.s001.pdf]

**Table A. Coefficients of the stiffness model for subject 1**

| Subject 1 | | | | | | | |
|---|---|---|---|---|---|---|---|
| Anchor point level | | Waist | | | | | |
| | | 1 | 2 | 3 | 1 | 2 | 3 |
| Thigh | 1 | 8.74E+01 | 8.17E+01 | 5.92E+01 | 1.18E-02 | 1.16E-02 | 2.88E-02 |
| | 2 | 8.10E+01 | 7.25E+01 | 6.35E+01 | 1.48E-02 | 1.73E-02 | 2.25E-02 |
| | 3 | 7.70E+01 | 8.77E+01 | 7.40E+01 | 1.56E-02 | 1.11E-02 | 1.73E-02 |
| Coefficient | | $C_1$ | | | $C_2$ | | |

**Table B. Coefficients of the stiffness model for subject 2**

| Subject 2 | | | | | | | |
|---|---|---|---|---|---|---|---|
| Anchor point level | | Waist | | | | | |
| | | 1 | 2 | 3 | 1 | 2 | 3 |
| Thigh | 1 | 8.91E+01 | 7.40E+01 | 5.43E+01 | 1.11E-02 | 1.38E-02 | 2.60E-02 |
| | 2 | 8.39E+01 | 7.52E+01 | 6.06E+01 | 1.26E-02 | 1.56E-02 | 2.48E-02 |
| | 3 | 9.09E+01 | 7.80E+01 | 6.79E+01 | 1.21E-02 | 1.46E-02 | 2.03E-02 |
| Coefficient | | $C_1$ | | | $C_2$ | | |

**Table C. Coefficients of the stiffness model for subject 3**

| Subject 3 | | | | | | | |
|---|---|---|---|---|---|---|---|
| Anchor point level | | Waist | | | | | |
| | | 1 | 2 | 3 | 1 | 2 | 3 |
| Thigh | 1 | 7.27E+01 | 7.24E+01 | 7.15E+01 | 1.86E-02 | 1.80E-02 | 1.68E-02 |
| | 2 | 7.68E+01 | 7.28E+01 | 7.58E+01 | 1.55E-02 | 1.72E-02 | 1.57E-02 |
| | 3 | 8.64E+01 | 8.32E+01 | 7.72E+01 | 1.19E-02 | 1.36E-02 | 1.54E-02 |
| Coefficient | | $C_1$ | | | $C_2$ | | |

**Table D. Coefficients of the stiffness model for subject 4**

| Subject 4 | | | | | | | |
|---|---|---|---|---|---|---|---|
| Anchor point level | | Waist | | | | | |
| | | 1 | 2 | 3 | 1 | 2 | 3 |
| Thigh | 1 | 7.28E+01 | 7.40E+01 | 6.03E+01 | 1.50E-02 | 1.33E-02 | 1.76E-02 |
| | 2 | 9.82E+01 | 1.05E+02 | 7.98E+01 | 9.90E-03 | 9.49E-03 | 1.29E-02 |
| | 3 | 1.02E+02 | 1.03E+02 | 8.27E+01 | 9.12E-03 | 8.41E-03 | 1.27E-02 |
| Coefficient | | $C_1$ | | | $C_2$ | | |

**Table E. Coefficients of the stiffness model for subject 5**

| Subject 5 | | | | | | | |
|---|---|---|---|---|---|---|---|
| Anchor point level | | Waist | | | | | |
| | | 1 | 2 | 3 | 1 | 2 | 3 |
| Thigh | 1 | 8.28E+01 | 7.18E+01 | 5.65E+01 | 1.15E-02 | 1.49E-02 | 2.41E-02 |
| | 2 | 8.90E+01 | 8.85E+01 | 6.73E+01 | 1.23E-02 | 1.14E-02 | 1.67E-02 |
| | 3 | 9.54E+01 | 1.04E+02 | 7.85E+01 | 1.05E-02 | 8.66E-03 | 1.51E-02 |
| Coefficient | | $C_1$ | | | $C_2$ | | |

**Table F. Coefficients of the stiffness model for subject 6**

| Subject 6 | | | | | | | |
|---|---|---|---|---|---|---|---|
| Anchor point level | | Waist | | | | | |
| | | 1 | 2 | 3 | 1 | 2 | 3 |
| Thigh | 1 | 9.71E+01 | 7.49E+01 | 4.99E+01 | 1.29E-02 | 1.89E-02 | 4.67E-02 |
| | 2 | 9.77E+01 | 8.38E+01 | 5.84E+01 | 1.19E-02 | 1.71E-02 | 3.52E-02 |
| | 3 | 9.72E+01 | 9.61E+01 | 6.03E+01 | 1.21E-02 | 1.34E-02 | 3.52E-02 |
| Coefficient | | $C_1$ | | | $C_2$ | | |

**Table G. Coefficients of the stiffness model for subject 7**

| Subject 7 | | | | | | | |
|---|---|---|---|---|---|---|---|
| Anchor point level | | Waist | | | | | |
| | | 1 | 2 | 3 | 1 | 2 | 3 |
| Thigh | 1 | 7.67E+01 | 7.54E+01 | 4.97E+01 | 1.39E-02 | 1.26E-02 | 2.66E-02 |
| | 2 | 8.35E+01 | 7.67E+01 | 5.81E+01 | 1.25E-02 | 1.33E-02 | 2.30E-02 |
| | 3 | 8.41E+01 | 7.16E+01 | 6.21E+01 | 1.20E-02 | 1.61E-02 | 2.16E-02 |
| Coefficient | | $C_1$ | | | $C_2$ | | |

**Table H. Coefficients of the stiffness model for subject 8**

| Subject 8 | | | | | | | |
|---|---|---|---|---|---|---|---|
| Anchor point level | | Waist | | | | | |
| | | 1 | 2 | 3 | 1 | 2 | 3 |
| Thigh | 1 | 7.26E+01 | 6.94E+01 | 6.41E+01 | 2.31E-02 | 2.10E-02 | 2.54E-02 |
| | 2 | 7.07E+01 | 8.17E+01 | 6.75E+01 | 2.02E-02 | 1.60E-02 | 2.10E-02 |
| | 3 | 8.08E+01 | 8.57E+01 | 6.44E+01 | 1.75E-02 | 1.61E-02 | 2.36E-02 |
| Coefficient | | $C_1$ | | | $C_2$ | | |
